# A Comprehensive Method for GNSS Data Quality Determination to Improve Ionospheric Data Analysis

**DOI:** 10.3390/s140814971

**Published:** 2014-08-14

**Authors:** Minchan Kim, Jiwon Seo, Jiyun Lee

**Affiliations:** 1 Division of Aerospace Engineering, Korea Advanced Institute of Science and Technology, 291 Daehak-ro, Daejeon 305-701, Korea; E-Mail: alsckszz@kaist.ac.kr; 2 School of Integrated Technology, Yonsei University, 85 Songdogwahak-ro, Incheon 406-840, Korea; E-Mail: jiwon.seo@yonsei.ac.kr; 3 Yonsei Institute of Convergence Technology, Yonsei University, 85 Songdogwahak-ro, Incheon 406-840, Korea

**Keywords:** GNSS reference stations, GNSS observations, quality determination, data quality parameters

## Abstract

Global Navigation Satellite Systems (GNSS) are now recognized as cost-effective tools for ionospheric studies by providing the global coverage through worldwide networks of GNSS stations. While GNSS networks continue to expand to improve the observability of the ionosphere, the amount of poor quality GNSS observation data is also increasing and the use of poor-quality GNSS data degrades the accuracy of ionospheric measurements. This paper develops a comprehensive method to determine the quality of GNSS observations for the purpose of ionospheric studies. The algorithms are designed especially to compute key GNSS data quality parameters which affect the quality of ionospheric product. The quality of data collected from the Continuously Operating Reference Stations (CORS) network in the conterminous United States (CONUS) is analyzed. The resulting quality varies widely, depending on each station and the data quality of individual stations persists for an extended time period. When compared to conventional methods, the quality parameters obtained from the proposed method have a stronger correlation with the quality of ionospheric data. The results suggest that a set of data quality parameters when used in combination can effectively select stations with high-quality GNSS data and improve the performance of ionospheric data analysis.

## 1. Introduction

Global Navigation Satellite Systems (GNSS) signals are measurably delayed as they pass through the Earth's ionosphere. Because the ionosphere is a dispersive medium, dual frequency GNSS observations can be utilized to generate Total Electron Content (TEC) estimates and thus contribute to various ionospheric studies. These include: modeling the ionosphere [1,2]; mapping global and local ionospheric TEC [3,4]; and studying ionospheric deformation caused by natural hazards such as earthquakes, volcanic eruptions, and tsunamis [5–8]. Recently ionospheric anomalies which can threaten navigation integrity of GNSS augmentation systems have also been extensively studied [9].

To be used in these various arenas, it is necessary to compute consistent high precision ionospheric measurements such as ionospheric delay on GNSS signal, ionospheric spatial gradient, and TEC using dual-frequency code and carrier measurements collected from GNSS reference station networks. However, in GNSS data processing, poor-quality GNSS observations can deteriorate the quality of ionospheric measurements. A discontinuity (*i.e.*, data jump) in GNSS observations can occur as a result of a cycle slip, that is, a sudden jump of a number of integer cycles in carrier-phase measurements. Cycle slips are caused by the loss of lock of receiver phase lock loops due to a receiver/antenna being shaded in an “urban canyon” or foliated environments, low Signal-to-Noise Ratio (SNR) of satellite signals, or failure from electromagnetic interference in the receiver itself. Multipath caused by the reflection of satellite signals from the ground, buildings, or other obstacles incurs rapidly changing errors in GNSS observations. Large multipath errors on the code measurements when used to level the carrier measurements introduce errors on the delay estimates. Short arcs caused by cycle slips, outliers, or invalid observations are typically subjected to large leveling errors and thus make poor delay estimates [10]. While these errors are detected, removed, and controlled in data pre-processing, they cause estimation errors on ionospheric measurements since the treatment cannot be perfect especially when the GNSS data quality is very poor.

Recently, GNSS networks have become more widespread around the world and the number of stations has increased, which has led to an improvement in the observability of ionospheric behaviors. The Continuously Operating Reference Stations (CORS) network has over 2100 stations as of 2013 in the conterminous United States (CONUS) compared to about 400 stations prior to 2004. Japan has one of the most densely populated Global Positioning System (GPS) networks, with the GPS Earth Observation Network (GEONET) consisting of over 1200 stations. The increase in the total number of stations has led to a corresponding increase in the number of station with poor GNSS data quality as well. The use of poor quality data degrades the accuracy of ionospheric measurements. Therefore it is necessary to have a tool to characterize the quality of GNSS data collected from stations. If the stations with high quality data are effectively selected by using quality information of those stations, it would help obtain more reliable results from ionospheric data analysis.

The most commonly used software tool to solve many pre-processing problems with GNSS is Translation, Editing, and Quality Checking (TEQC) [11]. This freeware program developed by the University NAVSTAR Consortium (UNAVCO) facility in Boulder (CO, USA) provides data quality information about the receiver clock slips, receiver cycle slips, multipath, receiver SNR, and other useful parameters and tracking statistics. The Leica GNSS Quality Control (Leica GNSS QC) software developed by Leica Geosystems also performs automatic quality checking and reporting of logged Receiver INdependent EXchange (RINEX) data [12]. Leica GNSS QC not only examines the quality (tracking information, data gaps, cycle slips, SNR, and multipath) of the data, but also graphically displays the multipath on code measurements, SNR, and coordinate information. While these programs support various options and provide general quality parameters, the programs do not target on providing the quality parameters which affect the quality of ionospheric product the most. Small cycle slips which cannot be detected by a conventional criterion (e.g., a threshold of 2 meters in TEQC [11]) may degrade the accuracy of ionospheric measurements if such event occurs frequently. The information about short arcs or outliers, not included in the freeware tools, could also be important because these events typically cause large leveling errors in ionospheric measurements [10]. The quality parameters which can be used to select GNSS observation data for ionospheric studies thus need to be carefully designed and the sensitivity of ionospheric data process to each parameter should be examined.

This paper presents a sophisticated data processing method to determine the quality of GNSS observations for the purpose of ionospheric studies. Section 2 discusses the effect of using poor quality GNSS data for ionospheric data analysis. Section 3 describes a series of algorithms which provides comprehensive and precise quality information about the cycle slips, short arcs, outliers, receiver noise, receiver SNR, multipath on L1 and L2 code measurements, and the daily number of observations. In Section 4, the data quality parameters of CORS stations within CONUS are analyzed. The correlation between the quality parameters and the quality of ionospheric data is also investigated. Section 5 concludes the paper with a discussion of the implications of this work.

## 2. Poor GNSS Data Quality and Its Effect

This section investigates the effect of GNSS data collected from stations with poor data quality on ionospheric measurements. Ionospheric delays and ionospheric spatial gradients are matters of concern for GNSS augmentation systems, such as Space-Based Augmentation Systems (SBAS) and Ground-Based Augmentation Systems (GBAS), because usually large spatial and temporal variations in ionospheric delays occurring during severe ionospheric storms could cause potential integrity threats to users [9]. Ground facilities of these systems monitor ionospheric anomalies defined by threat models and provide alarms to the users within time-to-alerts [13]. The ionospheric anomaly threat models are developed based on precise estimates of ionospheric delays and gradients computed using the dual-frequency GNSS reference network data of each region where systems are fielded [9].

GNSS observations are also used to compute the estimates of TEC and TEC perturbation in order to detect ionospheric disturbances caused by natural hazards including earthquakes, volcano eruptions, and tsunamis [14]. The natural hazards are known to generate electron density fluctuations in the ionosphere and TEC variations through atmospheric acoustic and gravity waves [6,8]. GNSS data are well suited to monitor ionospheric activity associated with natural hazards both in the locations of the occurrence and outside of it with sufficient spatial and temporal resolution to infer key properties such as velocity, direction, and magnitude of ionospheric disturbances. Because these applications use data collected from GNSS reference stations, the quality of the data from each station can affect the results of these studies.

**Figure 1.**
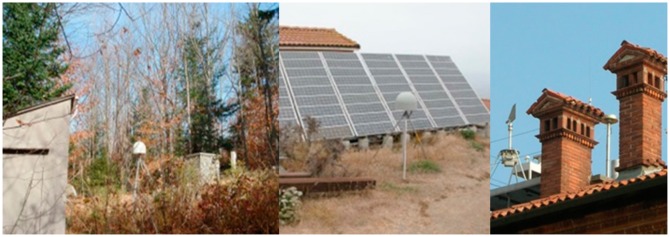
Examples of Poorly Sited CORS stations [15].

As shown in Figure 1, some stations of the CORS network in CONUS are actually located in unfriendly environments (e.g., next to a solar panel, in a bush, and even in-between towers [15]). Because signal loss and attenuation are induced by obstacles, such as metal plates and branches, raw GPS measurements from these stations are corrupted and consequently may produce erroneous estimates of ionospheric measurements.

**Figure 2.**
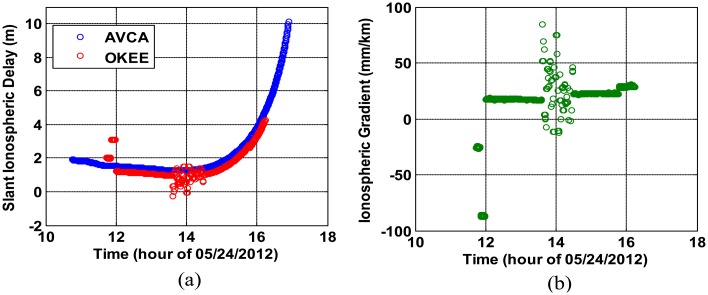
Examples of ionospheric measurements corrupted by poor quality GNSS data: (**a**) dual-frequency slant ionospheric delay estimates for CORS stations OKEE (poor quality data) and AVCA (good quality data) and (**b**) ionospheric spatial gradient estimates between OKEE and AVCA viewing PRN 22.

Figure 2a shows the ionospheric delay estimates of GPS L1 signals in the slant domain (*i.e.*, along the actual path between satellite and receiver) observed from two nearby stations OKEE and AVCA (separated by 18.01 km) while they tracked PRN 22 on a nominal day (24 May 2012). OKEE is a good example of a station with poor GPS data quality. From the many fragments of ionospheric delay estimates from OKEE (red), it is evident that its carrier-phase measurements are corrupted by numerous cycle slips resulting in outliers and short arcs of ionospheric observations. By dividing the differences in the ionospheric delays by the separation distance, the ionospheric spatial gradients between the two stations are estimated [16], as shown in Figure 2b. The ionospheric-delay leveling errors due to the short arcs from OKEE are observed at each end of the curve, and the many fragments due to the excessive cycle slips on OKEE are evident in the center of the curve.

**Figure 3.**
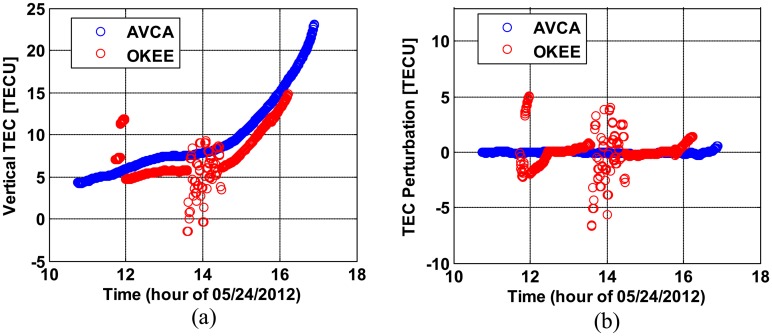
VTEC and VTEC perturbations corrupted by poor quality GNSS data: (**a**) dual-frequency vertical TEC estimates and (**b**) VTEC perturbations for CORS stations OKEE (poor quality data) and AVCA (good quality data).

Figure 3a,b shows vertical TEC (VTEC) estimates and VTEC perturbations of PRN 22 observed at OKEE and AVCA. TEC estimates in the slant domain were converted to equivalent vertical TEC (*i.e.*, in the zenith or 90-degree upward direction above the observing receiver) via a geometric mapping function [16]. The perturbations of VTEC were estimated by subtracting the large-scale trend of VTEC variation from the original VTEC [14]. The hourly trend of VTEC was estimated using a moving average filter with a time window of two hours. It is obvious in Figure 3b that the cycle clips and short arcs on OKEE cause large VTEC perturbations which are not real, because this rapid variation of VTEC is not expected under quiet conditions (see Table 1). These examples illustrate how poor data quality degrades the accuracy of ionospheric measurements and can produce erroneous results.

## 3. GNSS Data Quality Measurement Algorithms

**Figure 4.**
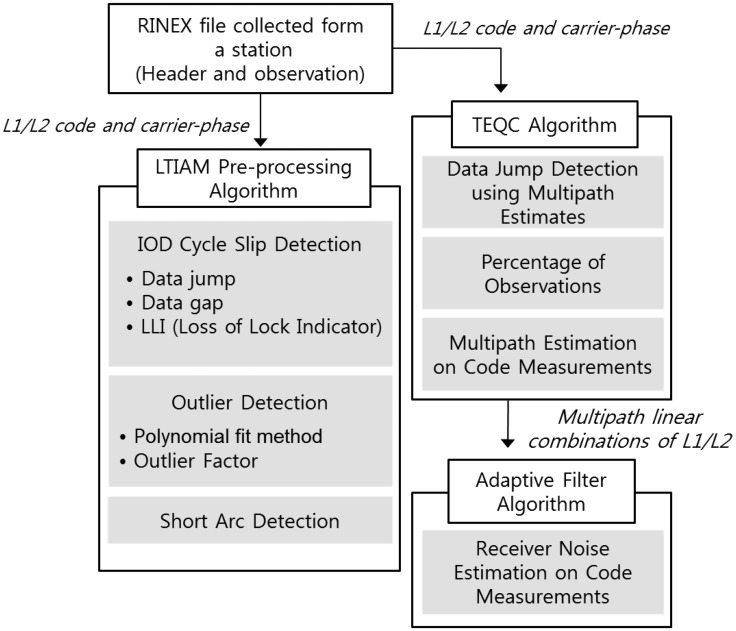
GNSS data quality measurement algorithms.

A methodology for determining the quality of GNSS data has been developed by utilizing the GNSS data pre-processing technique for ionospheric data analysis as a basis and augmenting it with the TEQC algorithm and adaptive filter algorithm [11,17–19]. The purpose of this method is to provide comprehensive and accurate quality information to users for the selection of high-quality GNSS data. The input of the method is the RINEX file collected from a station of our interest for two consecutive days and the output is the GNSS data quality parameters of the corresponding station. This method is composed of mainly three parts, as shown in Figure 4: Long-Term Ionospheric Anomaly Monitoring (LTIAM) pre-processing algorithm, TEQC algorithm, and adaptive filter algorithm.

The number of IOnospheric Delay (IOD) cycle slips, the number of outliers, and the number of short arcs are counted as separate data quality parameters using the LTIAM pre-processing algorithm. The percentage of observations, the Root Mean Square (RMS) of multipath on L1 code and L2 code measurements, and the number of data jumps detected using multipath estimates are computed by implementing the TEQC algorithm. Lastly, the mean of receiver noise on code measurements is calculated by designing an adaptive filter algorithm.

### 3.1. LTIAM Pre-Processing Algorithm

The LTIAM tool is an automated software package developed by the authors to build ionospheric anomaly threat models for GBAS and to evaluate the validity of the threat model over the life cycle of system by continually monitoring ionospheric behavior [17,18]. This tool automatically gathers GPS observation data during the potential days of anomalous ionospheric events which are selected based on external data from public space weather sites. After computing ionospheric delays and gradients using GPS data, the tool automatically searches for any anomalous gradients that are large enough to be potentially hazardous to GBAS users. The selected anomaly candidates will be manually validated and reported if deemed to be real anomalies.

High-quality ionospheric measurements are essential for the product of LTIAM. Thus, this tool includes a sophisticated pre-processing algorithm, which performs cycle slip detection, short arc removal, outlier removal, and code-carrier smoothing, to obtain precise estimates of ionospheric delays. In this paper, we utilize the existing LTIAM pre-processing algorithm to detect IOD cycle slips, outliers, and short arcs, and to count the numbers of these events as data quality parameters. The detection algorithms are described in Section 3.1.1, and detection thresholds are determined in Section 3.1.2.

#### 3.1.1. Detection of Cycle Slips, Outliers and Short Arcs

Cycle slip, outlier, and short arc detection methods have already been developed as a part of the LTIAM pre-processing algorithm. These detections are performed for each continuous arc of slant ionospheric delays estimated using dual-frequency carrier-phase measurements. The general forms of the GPS code (*ρ_L_*_1_, *ρ_L_*_2_) and carrier-phase measurements (*ϕ_L_*_1_, *ϕ_L_*_2_) for the L1 and L2 signal frequencies are expressed as:

(1) $$\rho_{L1}=r_{n}^{k}+I_{n}^{k}+M_{L1}+\varepsilon_{\rho 1}$$

(2) $$\rho_{L2}=r_{n}^{k}+\gamma I_{n}^{k}+M_{L2}+\varepsilon_{\rho 2}$$

(3) $$\phi_{L1}=r_{n}^{k}-I_{n}^{k}+N_{L1}+m_{L1}+\varepsilon_{\phi 1}$$

(4) $$\phi_{L2}=r_{n}^{k}-\gamma I_{n}^{k}+N_{L2}+m_{L2}+\varepsilon_{\phi 2}$$

(5) $$\gamma =\frac{f_{L1}^{2}}{f_{L2}^{2}}$$

The common term, $r_{n}^{k}$, represents the sum of the true range between the *n*th receiver and *k*th satellite, receiver clock biases, satellite clock biases, and tropospheric error. *N_Li_* is the integer ambiguity of the *Li* (*i* = 1, 2) frequency carrier-phase measurements. *M_Li_* and *m_Li_* are the multipath on code and carrier-phase measurements, respectively. The carrier-phase measurements have lower receiver noise errors than the code measurements (*i.e.*, ε*_ϕi_* ≪ ε_α_*_i_*). The ionospheric error, *I*, is of equal magnitude but opposite sign on the carrier phase relative to the code phase.

The slant ionospheric delay on the L1 signal at epoch *t_i_* and the difference of carrier-derived ionospheric delays between adjacent epochs, ∇*I_ϕ_*, are computed from the L1/L2 code (ρ*_L_*_1_, ρ*_L_*_2_) and carrier-phase (*ϕ_L_*_1_, *ϕ_L_*_2_) measurements as shown in Equations (6–8):

(6) $$I_{\rho }(t_{i})=\frac{\rho_{L2}(t_{i})-\rho_{L1}(t_{i})}{\gamma -1}=I(t_{i})+\frac{M_{L2}(t_{i})-M_{L1}(t_{i})}{\gamma -1}+\varepsilon_{\rho }(t_{i})$$

(7) $$I_{\phi }(t_{i})=\frac{\phi_{L1}(t_{i})-\phi_{L2}(t_{i})}{\gamma -1}=I(t_{i})+\frac{N_{L1}(t_{i})-N_{L2}(t_{i})}{\gamma -1}+\frac{m_{L1}(t_{i})-m_{L2}(t_{i})}{\gamma -1}+\varepsilon_{\phi }(t_{i})$$

(8) $$\nabla I_{\phi }=I_{\phi }\left(t_{i}\right)-I_{\phi }\left(t_{i-1}\right)$$

The dual-frequency code-derived estimate, *I_ρ_*, is noisier than the carrier-derived estimate, *I_ϕ_*, because the carrier-phase measurements have lower multipath and receiver noise errors than the code measurements (*i.e.*, *m_Li_* ≪ *M_Li_*, *ε_ϕi_* ≪ *ε_ρi_* (*i* =1, 2)).

Cycle slips detected by using ∇*I_ϕ_* are usually defined as “IOD cycle slips” [11]. The LTIAM IOD cycle detection algorithm performs better than the conventional data quality checking algorithm (e.g., TEQC, see Section 4) by applying three detection criteria: data jump, data gap, and loss of lock indicator. First, the difference between two adjacent ionospheric delays, ∇*I_ϕ_*, is examined to detect a data jump greater than the slip detection threshold of 0.5 m for a nominal day. The determination of the threshold is explained in the following subsection. Second, the absence of L1 or L2 carrier-phase measurements is considered as cycle slips. Third, the Loss of Lock Indicator (LLI) of each observation from raw GPS data in RINEX format is also utilized as an indicator of potential cycle slips.

After performing detection of cycle slips, continuous arcs are divided into several sub-arcs. The step of outlier detection is carried out for each sub-arc. Two approaches, the polynomial fit method and the adjacent point difference method, are executed in parallel to detect outliers [17,20]. First, a polynomial fit is performed on the carrier-derived ionospheric delay estimates, *I_ϕ_* , and the residuals (*i.e.*, the *I_ϕ_* data minus the polynomial fit, *P_fit_*), *R*, are computed for each epoch *t_i_* as shown in Equation (9). If the largest value of differential residuals, ∇*R* , between adjacent points exceeds an outlier detection threshold of 0.5 m, this point is classified as a potential outlier. The determination of the threshold is explained in the following subsection:

(9) $$R(t_{i})=I_{\phi }(t_{i})-P_{\text{fit}}(t_{i})$$

(10) $$\nabla R(t_{i})=R(t_{i})-R(t_{i-1})$$

Second, the Outlier Factor (OF) between adjacent points of the point *p* at time *t_p_* is computed as:

(11) $$OF(t_{p})=\underset{q\in \text{adjacent}}{\sum w_{pq}}\left|I_{p}-I_{q}\right|$$

(12) $$w_{\text{pq}}=\frac{1/\left|t_{p}-t_{q}\right|}{\sum_{r\in \text{adjacent}}1/\left|t_{p}-t_{r}\right|}$$

where *I_p_* and *I_q_* are *I_ϕ_* at time *t_p_* and *t_q_*, respectively [20]. *w* is the weight between two points, *p* and *q* at time *t_p_* and *t_q_*. In this equation, the set “adjacent” includes all points within fifteen minutes centered at the point *p* at time *t_p_*. If the potential outlier identified from the polynomial fit method returns the largest OF, the point is recorded as an outlier and removed. To detect all outliers, this process is repeated until no more outliers remain.

LTIAM also detects short arcs which are continuous arcs of less than ten data points or five minutes when an interval of data points is thirty seconds. The short arcs need to be discarded because leveling errors for those arcs are typically large and cause ionospheric delay estimation errors [17]. In this step, we count the number of IOD cycle slips, the number of outliers, and the number of short arcs as data quality parameters.

#### 3.1.2. Determination of Detection Thresholds

LTIAM was originally designed to process data from the period of anomalous ionospheric events. Thus, LTIAM pre-processing algorithms use relaxed detection thresholds in order to prevent ionospheric data from being misjudged as cycle slips or outliers and discarded under ionospheric storm conditions. A threshold of 2.5 m for cycle slip detection and a threshold of 0.8 m for outlier removal were used as defaults [17]. However, data quality checks are commonly conducted by using data from nominal days on which anomalous ionospheric events rarely happen. This section thus newly determines cycle slip and outlier detection thresholds respectively through statistical analyses. We first collect data from CORS stations in CONUS for seven consecutive days and obtain statistical distributions of differential ionospheric delays, ∇*I_ϕ_*, and differential residuals, ∇*R* (where the residuals, *R*, are the carrier-derived ionospheric delays, ***I_ϕ_***, minus the polynomial fit of *I_ϕ_*).

The geomagnetic conditions on these seven consecutive days are shown with two indices of global geomagnetic activity from space weather databases [21]: planetary K (Kp) and disturbance storm time (Dst). In this period, a total of 1654 CORS network stations were operating in CONUS. As Kp and Dst in Table 1 indicate, the geomagnetic storm condition was quiet. This allows CORS station data quality to be observed while minimizing any influence of abnormal ionospheric behavior.

**Figure 5.**
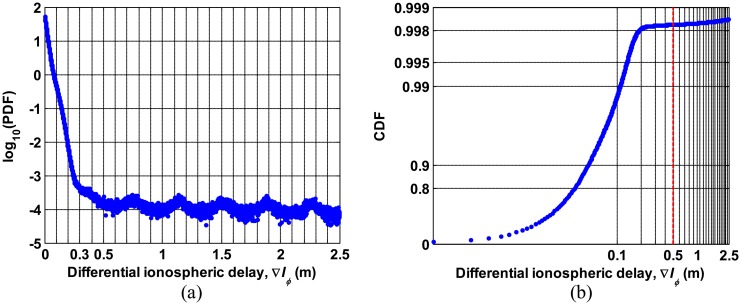
Distribution of differential ionosheric delays, ∇*I_ϕ_*, derived from data collected for seven consecutive days: (**a**) probability density function and (**b**) cumulative distribution function.

**Figure 6.**
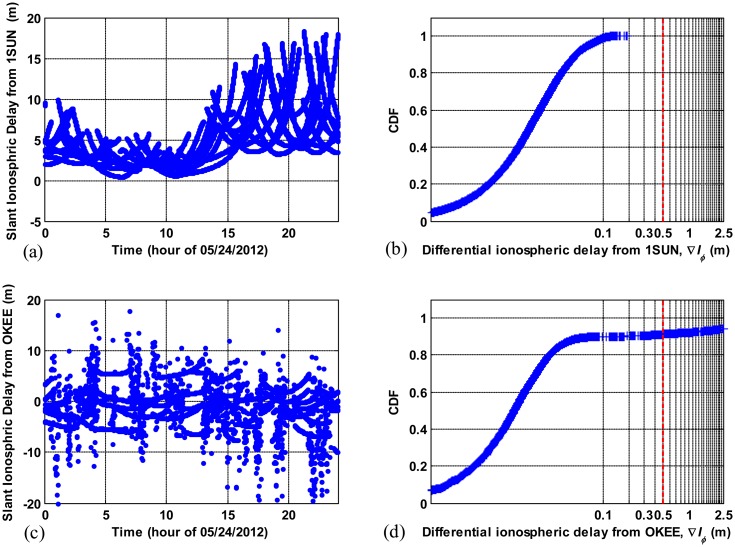
Example of ionospheric measurements corrupted by poor quality GNSS data: (**a**) dual-Frequency slant ionospheric delay estimates to all satellites for CORS station 1SUN (good quality data); (**b**) cumulative distribution function of differential ionosheric delay, ∇*I_ϕ_*, for 1SUN; (**c**) slant ionospheric delay estimates for CORS station OKEE (poor quality data); and (**d**) cumulative distribution function of differential ionosheric delay for OKEE.

Figure 5a,b shows the probability density function (PDF) on a logarithmic scale and cumulative distribution function (CDF) of ∇*I_ϕ_* derived from data for seven consecutive days respectively. In Figure 5a, we see that the PDF of ∇*I_ϕ_* steadily decreases as ∇*I_ϕ_* increases when ∇*I_ϕ_* is smaller than 0.5 meters, and PDF stays almost the same on the order of 10^−4^ for ∇*I_ϕ_* greater than 0.5 m. The CDF of ∇*I_ϕ_* in Figure 5b shows that the probability that ∇*I_ϕ_* goes beyond 0.5 meters is approximately 0.2 percent. This statistical result indicates that the rare occurrences of ∇*I_ϕ_* greater than 0.5 meters are likely to be due to cycle slips. One example of the comparison between erroneous ∇*I_ϕ_*and normal ∇*I_ϕ_* is shown in Figure 6. Figure 6a,c show the ionospheric delay estimates of all GPS satellites in the slant domain observed from stations 1SUN and OKEE on a nominal day (24 May 2012). The CDFs of ∇*I_ϕ_* derived from each station are shown in Figure 6b,d, respectively. The carrier-phase measurements of OKEE were corrupted by numerous cycle slips, resulting in inaccurate ionospheric delay estimates while good quality data of 1SUN produce precise ionospheric delay estimates. Approximately 10 percent of total ∇*I_ϕ_* of OKEE has a value greater than 0.5 m, while no ∇*I_ϕ_*from 1SUN exceeds 0.5 m. From these results, a threshold of 0.5 m was determined for cycle slip detection for nominal days.

**Figure 7.**
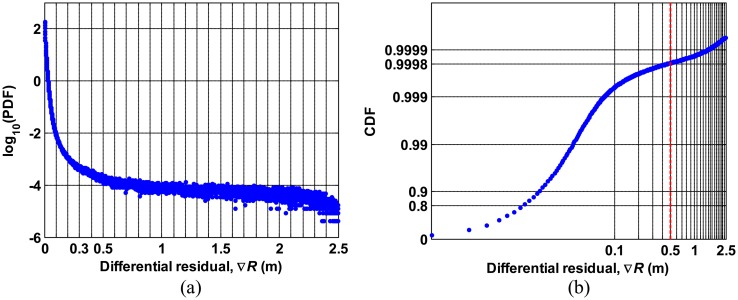
Distribution of differential residuals, ∇*R* , derived from data collected for seven consecutive days: (**a**) probability density function and (**b**) cumulative distribution function.

Figure 7a,b shows the PDF on a logarithmic scale and CDF of the differential residuals, ∇*R*, (where the residuals are the ionospheric delay data minus the polynomial fit) derived from data for seven consecutive days respectively. The distribution of ∇*R* shows that the probability is very small (on the order of 10^−4^) for ∇*R* greater than 0.5 m. As shown in Figure 7b, the probability that ∇*R* exceeds 0.5 m is approximately 0.02 percent. A threshold of 0.5 m is used for outlier detection in this study.

### 3.2. TEQC Algorithm

The TEQC software is commonly used to check data quality of GPS data in the RINEX format [11]. We selected and implemented some parts of TEQC algorithms to develop a comprehensive quality determination method for supporting broader communities including users for ionospheric studies. The quality parameters include the percentage of observations, the RMS of multipath on L1 and L2 code measurements, and the number of data jumps detected using multipath estimates. The percentage of observations is the ratio of “possible observations” to “complete observations,” where “possible observations” indicate the total number of possible observation epochs in a given time window, and “complete observations” are the number of epochs that actually observed code and carrier-phase data.

The LTIAM IOD cycle slip detection algorithm performs better than the IOD cycle slip detection of TEQC by applying three detection criteria. However, if data jumps occur in carrier-phase measurements due to receiver clock jumps (*i.e.*, receiver clock slips) on both L1 and L2 signals simultaneously, these cannot be detected using IOD measurements. Thus, we augmented slip detection by incorporating the TEQC method, which detects data jumps using multipath estimates. The data jumps detected by using multipath (MP) estimates are defined as MP slip. The MP slip method uses linear combinations of L1/L2 code (*ρ_L_*_1_, *ρ_L2_*) and carrier-phase (*ϕ_L_*_1_,*ϕ_L_*_2_) measurements [11]. These linear combinations are defined as:

(13) $$MP1\equiv \rho_{L1}-\left(1+\frac{2}{\gamma -1}\right)\phi_{L1}+\left(\frac{2}{\gamma -1}\right)\phi_{L2}=M_{L1}+B_{1}-\left(1+\frac{2}{\gamma -1}\right)m_{L1}+\left(\frac{2}{\gamma -1}\right)m_{L2}+\varepsilon_{1}$$

(14) $$MP2\equiv \rho_{L2}-\left(\frac{2\gamma }{\gamma -1}\right)\phi_{L1}+\left(\frac{2\gamma }{\gamma -1}-1\right)\phi_{L2}=M_{L2}+B_{2}-\left(\frac{2\gamma }{\gamma -1}\right)m_{L1}+\left(\frac{2\gamma }{\gamma -1}-1\right)m_{L2}+\varepsilon_{2}$$

*M_Li_* and *m_Li_* are the multipath errors on code and carrier-phase measurements on the *Li* (*i* = 1, 2) signals, respectively. The bias terms, *B*_l_ and *B*_2_, are:

(15) $$B_{1}\equiv -\left(1+\frac{2}{\gamma -1}\right)N_{L1}+\left(\frac{2}{\gamma -1}\right)N_{L2}$$

(16) $$B_{2}\equiv -\left(\frac{2\gamma }{\gamma -1}\right)N_{L1}+\left(\frac{2\gamma }{\gamma -1}-1\right)N_{L2}$$

*N_Li_* is the integer ambiguity of the *Li* frequency signals, and γ is the square of the frequency ratio as shown in Equation (5). When the difference between two consecutive points (at epoch *t_i_* and epoch *t_i_*_−1_) in each continuous arc of *MP*1 or *MP*2 is greater than a threshold of 10 m as shown in Equation (17), it is identified as a data jump. If the data jump occurs at a different point in time compared to an IOD cycle slip, this data jump is referred to as an MP slip:

(17) $$\left|MP1(t_{i})-MP1(t_{i-1})\right|>\text{threshold}$$

After performing IOD cycle slip and MP slip detection, the arcs are divided by the detected slips. The biases, *B*_1_ and *B*_2_, of the sub-arcs of *MP*1 and *MP*2 are assumed to be constants unless an undetected slip is remaining. Therefore, these constants are removed from each arc, and the RMS values of these linear combinations are reported. Although the portion of carrier-phase multipath is included in this reported value, the amount is small compared to that of code multipath. Thus, the bias-removed *MP*1 and *MP*2 can be approximated to be the multipath errors on L1 code and L2 code measurements, respectively.

### 3.3. Adaptive Filter Algorithm

An adaptive filter algorithm is designed to estimate receiver noise on code measurements. After removing the bias components, *B*_1_ and *B*_2_ , of *MP*1 and *MP*2 from Equations (13)–(16), *Mpi*_*new* can be expressed as:

(18) $$MP1\_\text{new}=MP1-B_{1}=mp_{1}+\varepsilon_{1}$$

(19) $$MP2\_\text{new}=MP2-B_{2}=mp_{2}+\varepsilon_{2}$$

(20) $$mp_{1}=M_{L1}-\left(1+\frac{2}{\gamma -1}\right)m_{L1}+\left(\frac{2}{\gamma -1}\right)m_{L2}$$

(21) $$mp_{2}=M_{L2}-\left(\frac{2\gamma }{\gamma -1}\right)m_{L1}+\left(\frac{2\gamma }{\gamma -1}-1\right)m_{L2}$$

*mp_i_*, the *Li*-frequency approximated code multipath estimate, is likely to be highly correlated to *mp_i_* from the previous day (*i.e.*, one sidereal day earlier). However, *ε_i_*, the receiver noise on *Li* code, is not correlated to *ε_i_* of the previous day. Therefore, *MPi*_*new* from two consecutive days can be separated into the correlated component (*mp_i_*) and the uncorrelated component (*ε_i_*) using an adaptive filter [19]. The adaptive filter takes two inputs: a primary input and a reference input. In this study, *Mpi*_*new* for the day of interest is set as the primary input, and *MPi*_*new* for the previous day is set as the reference input. Then, the output of a Finite-duration Impulse Response (FIR) filter is calculated using the reference input and weights. A least-mean-square (LMS) algorithm has been used to adaptively adjust the weights of the FIR filter to minimize the sum of squared estimation errors.

The adaptive filter returns the part of the primary input that is strongly correlated with the reference input as its output. Thus, the *mp_i_* of the primary input (*i.e.*, the multipath estimate on the code measurement) is calculated as the output of the adaptive filter. The estimation error of the filter approximately represents the code receiver noise, *ε_i_*, because it represents the value with *mp_i_* removed from the primary input. As explained, in order to estimate the receiver noise, *ε_i_*, correlation between the *Mpi* of two consecutive days must exist. However, there are cases where such correlation is not clearly visible depending on receiver/antenna type and environmental changes. In these cases, the receiver noise in the quality output is presented as “not available (N/A)”.

## 4. Results

The CORS data on the dates in Table 1 were collected and analyzed to evaluate the performance of the data quality measurement algorithms. As explained above, these seven consecutive days during which the geomagnetic storm condition was quiet are suitable for observing GNSS data quality because the chance of cycle slips and outliers being falsely detected due to any influence of abnormal ionospheric behavior is minimized. Using the results of this method, the comparative analysis on the performance of stations in the CORS network was conducted in Sections 4.1 and 4.2. In Section 4.3, we also examine the correlation between the data quality parameters obtained in this study and TEC perturbation which well represents the quality of ionospheric data under ionospherically quiet conditions. These results from correlation analysis are compared to that of the TEQC software. Section 4.4 discusses the selection of high quality data which can be conducted by utilizing data quality parameters through case studies.

### 4.1. Data Quality Parameter Output per Station

The statistics of quality parameters obtained from the tests are used to compare the performance of each station. Table 2 shows the results from the GNSS data quality measurement algorithms for station NVLA on 27 May 2012. The receiver model and the type of antenna can be found in the header part of the RINEX file collected from the station. While the RINEX file records the SNR for L1 and L2 frequencies, the unit of SNR is dependent on each receiver and not all stations provide SNR. Since the GPS observations at low elevation angles (*i.e.*, weaker received signal strengths) are affected by larger multipath errors and prone to loss of lock, an elevation cutoff angle of 10 degrees (as a default) is used. The number of IOD cycle slips, the number of outliers, and the number of short arcs, the percentage of observations, the number of MP slips, the RMS of multipath errors on L1 and L2 code measurements, and the mean of receiver noise on L1 and L2 code measurements are computed using the proposed data quality determination method.

The quality measurements corresponding to those in Table 2 are obtained from each station every day during the seven days listed in Table 1. Table 3 shows the rank of stations for five quality parameters (each parameter of stations is averaged over all seven days) among a total of thirteen parameters. The worst station is on the top for each quality parameter, and the same station is highlighted with the same color. Table 3 shows that the worst stations are likely to be identified by multiple data quality parameters. Recall that, among the highlighted stations in this table, station OKEE was introduced as an example of station with poor GPS data quality in Section 2.

### 4.2. Distributions of Data Quality Parameters

**Figure 8.**
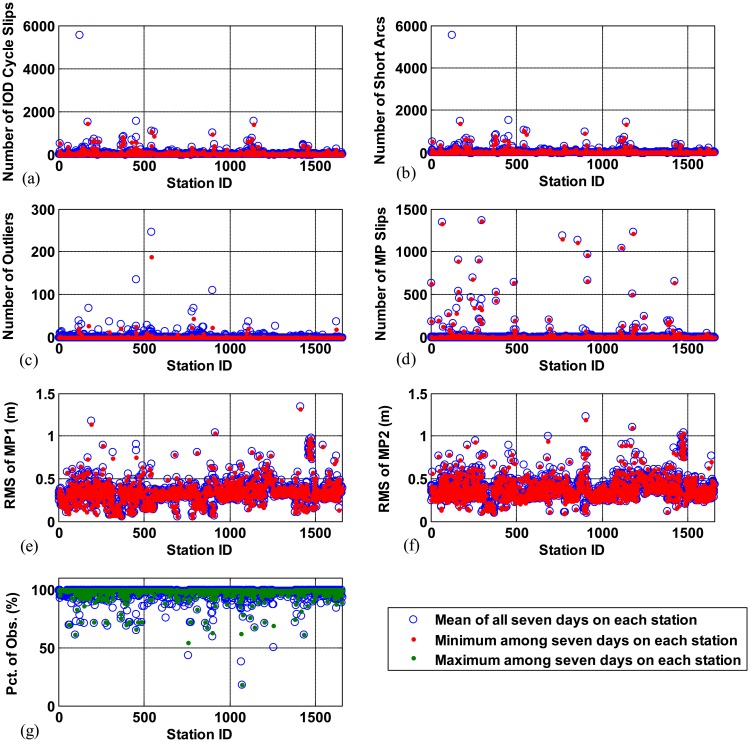
Data quality parameters obtained at each station per day: (**a**) number of IOD cycle slips; (**b**) number of short arcs; (**c**) number of outliers; (**d**) number of MP slips; (**e**) RMS of MP1; (**f**) RMS of MP2; and (**g**) percentage of observations.

The results of analyzing the quality parameters of the CORS stations in CONUS show us how widely station performance can vary. Figure 8a shows the total number of IOD cycle slips counted over all satellites during 24 h at each station. These numbers are counted for the seven consecutive days. The station ID is plotted (in no particular order) along the *x*-axis, and the number of IOD cycle slips is plotted along the *y*-axis. The blue circle shows the mean value of all seven days on each station and the red dot represents the minimum value among seven days on each station, respectively. These two values over seven days are close together for most stations, indicating that poor data quality of a station persists for an extended period. From this test, 1.2 percent of stations had more than 500 IOD cycle slips per day, and more than 15 percent of the stations had more than 50 IOD cycle slips. Note that the mean value over all seven days and all stations is 39.94.

Figure 8b shows the total number of short arcs counted over all satellites during 24 h at each station. If cycle slips frequently occur, the number of short arcs increases because ionospheric delay data are divided into sub-arcs by the cycle slips. Thus, a high correlation exists between the number of IOD cycle slips and the number of short arcs. More than 10 percent of the stations had more than 50 short arcs per day while the mean value over all seven days and all stations is 34.48. The number of outliers also widely varies depending on station performance as shown in Figure 8c. This quality parameter has the mean value of 1.21 over all days and all stations, and as many as 2.4 percent of stations had more than 10 outliers per day.

Figure 8d presents the total number of MP slips counted at each station per day. While the occurrence of IOD cycle slips mainly depends on environmental conditions around receivers, MP slips occurring due to receiver clock jumps rely upon the receiver itself. Thus, the distribution of the number of MP slips is dissimilar to that of IOD cycle slips. The mean value over all seven days and all stations is 15.02 MP slips per day, while one percent of stations had more than 500 MP slips per day, and more than 3.6 percent of the stations had more than 50 MP slips. The RMS values of multipath errors on L1 code and L2 code measurements of each station are shown in Figure 8e,f, respectively. Since multipath errors are caused by the reflection of satellite signals from the environment around receivers such as the ground, buildings, or other obstacles, the distributions of RMS multipath on L1 and L2 code measurements are very much alike. The mean values of RMS of multipath errors on L1 and L2 code measurements over all days and all stations are 0.3411 and 0.3876 m, respectively.

In most quality parameters, better data quality results in smaller values. However, higher values of percentage of observations indicate better quality of data. Thus, the mean values (blue circle) and the maximum values (green dots) of the percentage of observations of each station are compared to confirm that the poor data quality of a station persists for an extended period. In the percentage of observations, the maximum value (green dots) of a station across seven days and the mean value (blue circle) over seven days are also close together for most stations as shown in Figure 8g. The mean value of the percentage of observations over all days and all stations is 97.39 percent. As can be seen in Figure 8a–g, the range of good and poor performance varies noticeably for each quality parameter. It can be observed that most stations maintain similar performance for the duration of this data set. This information suggests that we can select high quality GNSS data in a station basis and the quality parameters of each station should be useful for the selection.

**Figure 9.**
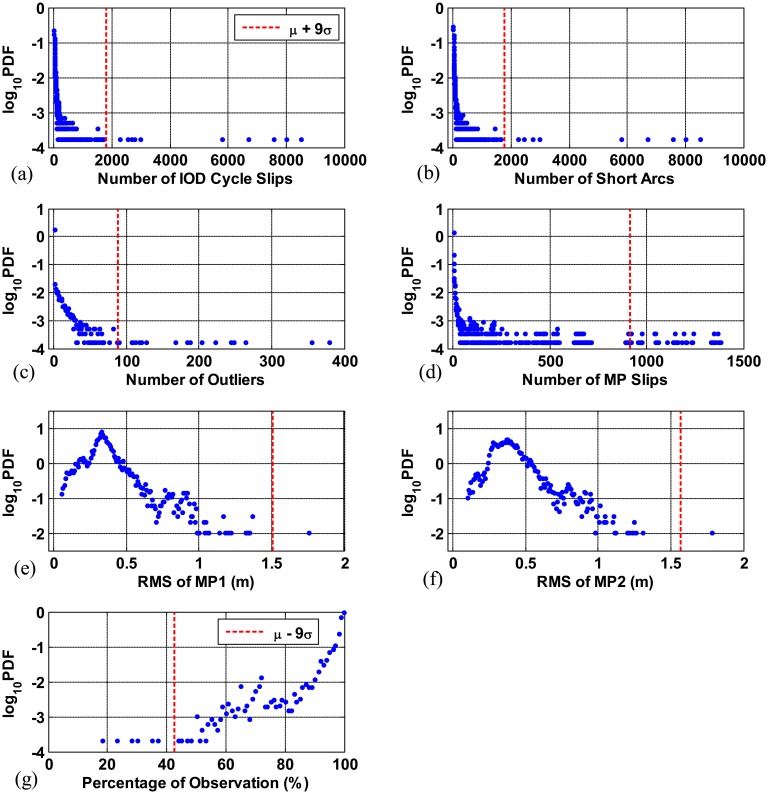
Probability density function of data quality parameter for each station per day (data collected for seven days): (**a**) number of IOD cycle slips; (**b**) number of short arcs; (**c**) number of outliers; (**d**) number of MP slips; (**e**) RMS of MP1; (**f**) RMS of MP2; and (**g**) percentage of observations.

Figure 9a through 9g show the PDF of each quality parameter on each station per day in logarithmic scale. These test statistics are obtained from data collected for the seven days in Table 1. As an example, the PDF of the number of IOD cycle slips on each station per day is shown in Figure 9a. The dashed vertical lines in Figure 9a–f refer to the value of *μ*+9*σ* (the mean value plus 9 times the sample standard deviation) for each parameter. In Figure 9a–f, since data (blue) exist continuously from 0 to this line and the continuity of data ceases beyond this line, the data that go beyond *μ*+9*σ* are considered to be extreme outliers (*i.e.*, stations with poor data quality). The dashed vertical line in Figure 9g represents the value of *μ*+9*σ*. The percentage of observations which falls lower than this line indicates extremely poor data quality.

### 4.3. Correlation between Data Quality Parameters and TEC Perturbations

**Figure 10.**
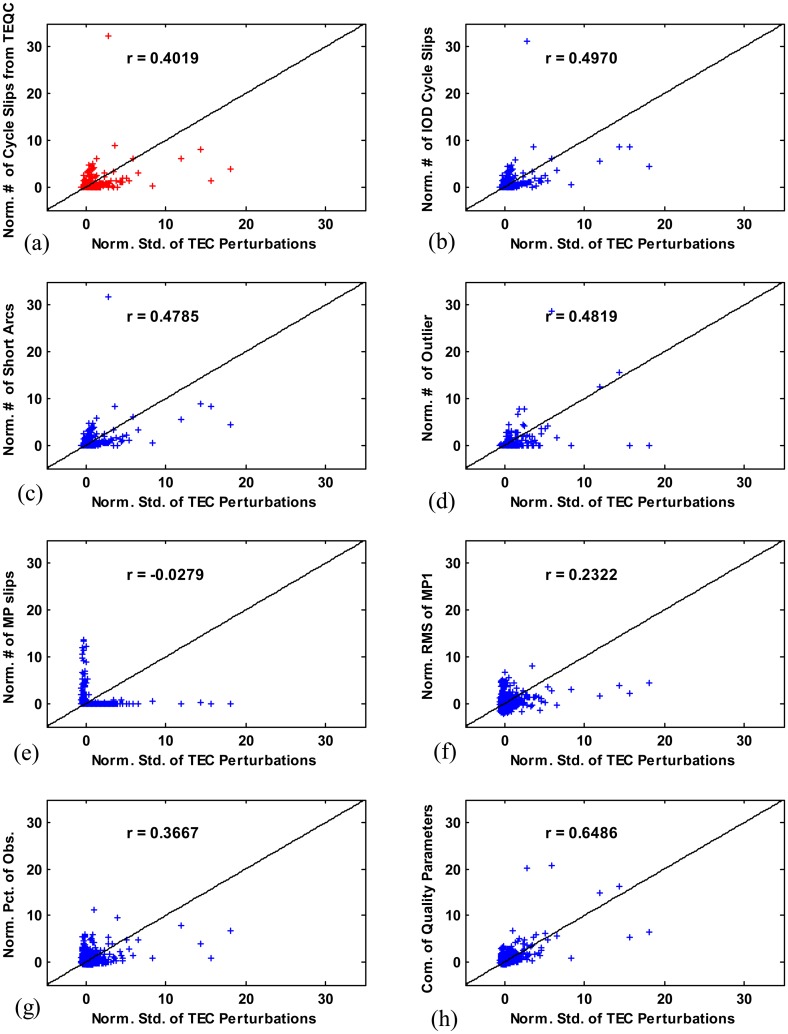
Correlation of TEC perturbation with data quality parameters for CORS stations (all parameters are normalized): (**a**) number of IOD cycle slips obtained from TEQC; (**b**) number of IOD cycle slips; (**c**) number of short arcs; (**d**) number of outliers; (**e**) number of MP slips; (**f**) RMS of MP1; (**g**) percentage of observations; and (**h**) combination of three quality parameters.

To examine the possibility of selecting high quality GNSS data (*i.e.*, high quality stations) based on data quality parameters for ionospheric studies, the correlation between TEC perturbation and each quality parameter was investigated. The TEC perturbation measurements are generated by processing dual-frequency GPS measurements collected from the CORS network using the LTIAM software. In Figure 10, normalized standard deviations of TEC perturbation calculated during the seven days listed in Table 1 are plotted along the *x*-axis. The standard deviations of TEC perturbation obtained from data over all satellites during 24 h at each station are averaged over the seven days. The averaged standard deviations of TEC perturbation at each station are normalized by removing their mean over all stations and dividing them by their standard deviations. Large TEC perturbation is not expected to be seen in mid latitude regions on the dates (listed in Table 1) during which geomagnetic activities were quiet. Thus the large TEC perturbations observed from some stations in Figure 10 are likely due to poor quality GPS data corrupted by cycle slips, outliers, multipath, and so on.

The correlation between TEC perturbation and the number of IOD cycle slips counted using the TEQC software tool is shown in Figure 10a. As done for the proposed method in this paper, TEQC also used differential ionospheric delay, ∇*I_ϕ_*, for cycle slip detection. To make a direct comparison of its performance to that of the proposed method (from which the results are shown in Figure 10b), we set a threshold of 0.5 m in TEQC which is the same value determined in Section 3.1.2. The number of cycle slips is counted using TEQC over all satellites during 24 h at each station. These numbers are averaged over the seven days and then normalized over all stations to have zero mean and unit variance. In this case, the Pearson's correlation coefficient between the number of cycle slips and the TEC perturbation, *r*, is 0.4019.

The six quality parameters (the number of IOD cycle slips, the number of short arcs, the number of outliers, the number of MP slips, the RMS of MP1, and the percentage of observation) obtained from the proposed method in this paper are calculated, averaged over seven days, normalized over all stations, and plotted along the *y*-axis in Figure 10b–g. As shown in Figure 10b, the TEC perturbation is more highly correlated with the number of IOD cycle slips (*r*= 0.4970) than with the number of cycle slips (*r*= 0.4019) derived from TEQC. This result demonstrates that the cycle slip detection of the proposed method performs better and its output more accurately represents GPS data quality. The correlation coefficients were also considerably high in the cases of the number of short arcs (*r* = 0.4785) and the number of outliers (*r* = 0.4891), indicating that these would affect the quality of ionospheric data. The correlations of TEC perturbation with MP1 and the percentage of observation are not strong, and that with the number of MP slips which are not visible on TEC estimates is weak. Assuming that the normalized quality parameters are independent and the sum of the normalized quality parameters has a normal distribution, we combine multiple quality parameters into a new quality parameter which has a stronger correlation with TEC perturbation. As shown in Figure 10h, the correlation increased (*r*= 0.6486) after adding the three quality parameters which have the first to third highest correlation: the number of IOD cycle slip, the number of outliers, and percentage of observation. In this combination set, the number of short arcs was not included to avoid adding duplicated information because its distribution is almost equal to that of the number of IOD cycle slips. More effective station selection would be possible using the parameter with higher correlation (which will be discussed in the following subsection).

### 4.4. Case Study: Station Selection

**Figure 11.**
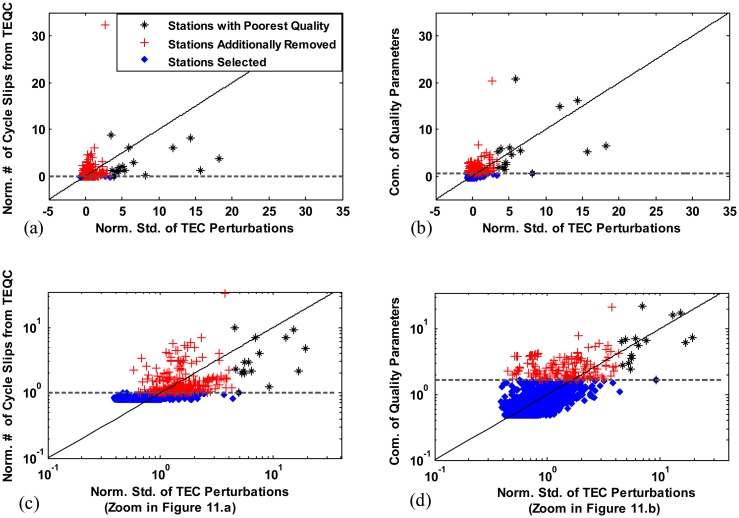
Station selection using: (**a**) the number of IOD cycle slips generated using TEQC; (**b**) combination of three quality parameters determined using the proposed method; (**c**) zoomed in (**a**); and (**d**) zoomed in (**b**) in a logarithmic scale.

This subsection shows the possibility of utilizing the data quality parameters driven by the proposed method to select stations with high quality GNSS data. The performances of two cases which use the TEQC-driven cycle slip parameter and the combined quality parameter respectively were compared. Figure 10a,h which present results from correlation analyses for the two parameters were redrawn in Figure 11a,b. Based on the standard deviation of TEC perturbation, sixteen stations (1 percent of the total stations) that produce ionospheric data with the poorest quality were identified and denoted with black asterisks in Figure 11a–d. To exclude these worst case stations using the number of cycle slips obtained from TEQC, the threshold of the normalized number of cycle slips is lowered to 0.0074 (demarcated with the dashed horizontal line in Figure 11a). However, if we select stations that fall below the threshold, 286 stations denoted with red crosses (17 percent of the total stations) in Figure 11a are sacrificed although these stations have good quality data. On the other hand, in the case of using the newly defined parameter by combining three quality parameters which have the highest correlation coefficients, only 164 (10 percent of the total stations) stations marked with red crosses in Figure 11b are additionally removed because of exceeding a threshold of 0.6469 (demarcated with the dashed horizontal line in Figure 11b). The stations marked with blue diamonds are selected as shown in Figure 11c,d (the zoomed-in plots of Figure 11a,b). Note that a value of one was added to all of the data prior to plotting, because negative data cannot be represented in a logarithmic scale. It is evident that the use of parameter which has stronger correlation with the quality of ionospheric data improves the performance of station selection. The set of data quality parameters when used in combination allowed the effective selection of high quality GNSS data and better performance compared to the TEQC parameter, although this is not necessarily the best solution. Research on the optimal means of utilizing data quality parameters generated by the proposed method for selecting high quality stations is in progress [22] and beyond the scope of this paper.

## 5. Conclusions

The use of corrupted GNSS data degrades the quality of ionospheric measurements. Thus, it is necessary to check the quality of observation data and use high quality GNSS data only for ionospheric data analysis. This paper presents a methodology to determine the quality of GNSS observations collected from a reference station for the purpose of ionospheric studies. This method provides a comprehensive set of quality control parameters calculated using the sophisticated pre-processing algorithms of the LTIAM which are augmented with the TEQC algorithm and adaptive filter algorithm. These quality parameters include the number of cycle slips, the number of short arcs, the number of outliers, the number of MP slips, the percentage of observations, the RMS of multipath on L1 and L2 code measurements, and the mean of receiver noise. The results from analyzing the GNSS data quality of the CORS network showed that the range of good and poor qualities varies noticeably for each quality parameter and the performance of individual stations persists for an extended time period. This indicates that high quality data can be selected in a station basis by utilizing data quality parameters. The correlation analysis between data quality parameters and TEC perturbations which well represent the quality of ionospheric data demonstrated that the quality parameters obtained from proposed method have stronger correlation than that of TEQC and thus enable a better performance when used for station selection. Furthermore, a set of quality parameters was used in combination, its correlation with TEC perturbations increased and the performance of selecting high quality stations was improved.

As the number of GNSS stations and also GNSS applications where their observations can be employed steadily increase, it becomes more important to characterize the quality of GNSS observations. The proposed method should be applicable for the GNSS users of various applications to check the quality of GNSS observations and accordingly select high-quality data. This will especially help to improve the performance of applications for which precise GNSS data is essential, such as Real Time Kinematic (RTK), precise orbit determination of satellites, and the estimation of the Earth Rotation Parameters (ERP). The use of the statistical information on quality parameters obtained from this method allows selecting stations desired for specific applications. Research on the best means of utilizing these statistical results and effectively selecting stations with high quality data is an ongoing research topic that will benefit a wide range of GNSS applications.

## Figures and Tables

**Table 1.** Dates analyzed to determine detection thresholds.

Day (UT dd/mm/yy)	Kp	Dst
24/05/12	2.0	−15
25/05/12	2.3	17
26/05/12	2.3	−6
27/05/12	1.3	14
28/05/12	2.3	23
29/05/12	2.3	23
30/05/12	2.3	16

**Table 2.** Information of data quality parameters for Station NVLA on 27 May 2012.

Output Parameters	Example	Description
Date	27 May 2012	Day Month Year
Station ID	NVLA	
Receiver type	LEICA GRX1200PRO	
Antenna type	LEIAT504	
Possible observations (>10°)	26,553	Total number of possible observation epochs in a given time window
Complete observations (>10°)	25,223	Number of epochs that actually had L1/L2 code and carrier-phase data from at least one SV.
Percentage of observations	95	(Complete observations/possible observations) × 100
Mean S1 (>10°)	46.39	Mean signal to noise ratio (SNR) for L1
Mean S2 (>10°)	42.27	Mean signal to noise ratio (SNR) for L2
IOD cycle slips (>10°)	61	Total number of ionospheric delay (IOD) cycle slips occurred
MP slips (>10°)	0	Total number of multipath (MP) slips occurred
Outliers (>10°)	0	Total number of outliers observed
Short arcs (>10°)	40	Total number of short arcs observed
RMS MP1 (>10°)	0.3759 (m)	RMS of multipath on L1 code measurements
RMS MP2 (>10°)	0.3938 (m)	RMS of multipath on L2 code measurements
Receiver noise1 (>10°)	0.0808 (m)	Mean of receiver noise on L1 code measurements
Receiver noise2 (>10°)	0.1046 (m)	Mean of receiver noise on L2 code measurements

**Table 3.** Rank of CORS stations in CONUS (Worst station is on top for each quality parameter).

	# of IOD Cycle Slips		# of Short Arcs		Pct. of Obs.		# of Outliers		RMS of MP1	
Rank	Stn.	#	Stn.	#	Stn.	%	Stn.	#	Stn.	meter
1	bru5	5552.00	bru5	5545.14	p702	18.00	mion	246.14	wach	1.3460
2	ls02	1565.50	ls02	1559.16	p699	38.33	ls02	135.00	defi	1.1764
3	sag5	1544.00	covx	1484.85	ncwj	42.85	okee	109.29	ormd	1.0433
4	covx	1531.71	sag5	1466.42	twhl	50.71	cpac	68.29	zoa2	0.9758
5	mion	1100.86	mion	1064.71	okee	59.71	njwc	67.14	zfw1	0.9606
6	mlf5	1063.86	mlf5	1051.43	barn	61.00	njcm	58.86	zla1	0.9397
7	okee	1024.14	okee	1009.43	wvbr	61.00	brtw	38.71	zma1	0.9198
8	kns6	862.42	kns6	862.14	loz1	64.85	hruf	37.57	zau1	0.9197
9	loz1	832.42	kew6	819.57	ohfa	67.00	pltk	37.29	zob1	0.9143
10	kew6	819.71	loz1	793.71	sag6	67.00	p671	35.86	loz1	0.9100
11	red6	767.57	red6	760.14	hgis	68.85	jxvl	30.57	zse1	0.9086
12	drv6	715.14	drv6	705.85	kysc	68.85	ccgn	30.43	nas0	0.8977
13	lou6	673.71	lou6	646.71	arlr	70.00	mihl	27.71	zlc1	0.8975
14	prry	642.28	prry	619.28	arm3	70.00	lpsb	26.29	zmp1	0.8974
15	frtg	637.14	det6	617.86	dqcy	71.14	txbk	25.29	gol2	0.8972
16	plo5	625.57	plo5	616.00	hamm	71.14	nypb	24.71	zoa1	0.8841
17	det6	621.85	frtg	610.57	oakh	71.29	pbch	24.71	zdv1	0.8703
18	kew5	579.29	kew5	574.57	thhr	71.43	bnfy	24.14	zab1	0.8624
19	cosa	572.14	acu5	537.00	chzz	71.50	nyqn	24.14	zab2	0.8455
20	acu5	541.43	kns5	483.00	lsua	71.57	njgt	21.29	ls02	0.8386
